# Comparison of 2 models for gene–environment interactions: an example of simulated gene–medication interactions on systolic blood pressure in family-based data

**DOI:** 10.1186/s12919-016-0058-1

**Published:** 2016-10-18

**Authors:** Lindsay Fernández-Rhodes, Chani J. Hodonsky, Mariaelisa Graff, Shelly-Ann M. Love, Annie Green Howard, Amanda A. Seyerle, Christy L. Avery, Geetha Chittoor, Nora Franceschini, V. Saroja Voruganti, Kristin Young, Jeffrey R. O’Connell, Kari E. North, Anne E. Justice

**Affiliations:** 1 Department of Epidemiology, University of North Carolina at Chapel Hill, Chapel Hill, NC 27514 USA; 2 Department of Biostatistics, University of North Carolina at Chapel Hill, Chapel Hill, NC 27514 USA; 3 Department of Nutrition, and UNC Nutrition Research Institute, University of North Carolina, Kannapolis, NC 28081 USA; 4 School of Medicine, University of Maryland, Baltimore, MD 21201 USA

## Abstract

**Background:**

Nearly half of adults in the United States who are diagnosed with hypertension use blood-pressure-lowering medications. Yet there is a large interindividual variability in the response to these medications. Two complementary gene–environment interaction methods have been published and incorporated into publicly available software packages to examine interaction effects, including whether genetic variants modify the association between medication use and blood pressure. The first approach uses a gene–environment interaction term to measure the change in outcome when both the genetic marker and medication are present (the “interaction model”). The second approach tests for effect-size differences between strata of an environmental exposure (the “med-diff” approach). However, no studies have quantitatively compared how these methods perform with respect to 1 or 2 degree of freedom (DF) tests or in family-based data sets. We evaluated these 2 approaches using simulated genotype–medication response interactions at 3 single nucleotide polymorphisms (SNPs) across a range of minor allele frequencies (MAFs 0.1–5.4 %) using the Genetic Analysis Workshop 19 family sample.

**Results:**

The estimated interaction effect sizes were on average larger in the interaction model approach compared to the med-diff approach. The true positive proportion was higher for the med-diff approach for SNPs less than 1 % MAF, but higher for the interaction model when common variants were evaluated (MAF >5 %). The interaction model produced lower false-positive proportions than expected (5 %) across a range of MAFs for both the 1DF and 2DF tests. In contrast, the med-diff approach produced higher but stable false-positive proportions around 5 % across MAFs for both tests.

**Conclusions:**

Although the 1DF tests both performed similarly for common variants, the interaction model estimated true interaction effects with less bias and higher true positive proportions than the med-diff approach. However, if rare variation (MAF <5 %) is of interest, our findings suggest that when convergence is achieved, the med-diff approach may estimate true interaction effects more conservatively and with less variability.

## Background

Hypertension—defined as an average systolic blood pressure (SBP) of 140 mm Hg or higher or an average diastolic blood pressure (DBP) of 90 mm Hg or higher—affects approximately 30 % of American adults, 45 % of whom use antihypertensive medications for blood pressure (BP) control [1, 2]. Broad interindividual variability in responsiveness to antihypertensive medications suggests that genetics may modify response to treatment [3, 4]. Furthermore, SBP and DBP are heritable, and candidate-gene and genome-wide association studies have uncovered more than 50 loci associated with BP [5–15]. Detection of genetic markers responsible for differential pharmacologic response inform our understanding of biological pathways relevant to hypertension, as well as future interventions to reduce its burden [16, 17].

Two complementary gene–environment (G × E) interaction methods have been described in the literature to test G × E interactions such as differential response to antihypertensives resulting from genetic variation. The first method (the “interaction model”) tests for interaction using a gene–environment interaction term to measure the change in outcome when both the genetic marker and environmental factor are present, as compared to when the genetic marker is present but the environmental factor is not [18]. The second method (the “med-diff” approach) tests for effect size differences between strata that differ by environmental exposure [19]. Both methods can estimate 1 degree of freedom (DF) tests of gene-medication interactions as well as 2DF (or joint) tests of these interactions and the genetic main effect using publicly available software.

Although these methods have been assumed to be theoretically equivalent, no previous studies have directly compared them. Therefore, in this study we aimed to evaluate their performance by comparing both their power to detect simulated interaction effects as well as their false-positive proportions (FPPs) in family-based data from the Genetic Analysis Workshop 19 (GAW19) [20]. This was done by first calculating the true-positive proportion (TPP) for the 1DF and 2DF tests using 3 coding variants at *CYP3A43* of varying minor allele frequencies (MAFs) with simulated genotype–medication response interactions. We then used TPP to evaluate the power to detect simulated main effects at *MAP4* (the simulated single nucleotide polymorphisms, SNPs, with the largest proportion of variance explained in SBP, MAF 2.7 %) using a 2DF test in each approach. Lastly, we assessed the observed FPPs of each approach across the odd-numbered chromosomes without simulated effect using both 1DF and 2DF tests using publicly available software.

## Methods

Type 2 Diabetes Genetic Exploration by Next-generation sequencing in Ethnic Samples (T2D-GENES) Consortium Project [21] genotypic and GAW19 simulated phenotypic data have been described separately [20]. The GAW19 genotypic dosage data come from whole genome sequence variants for 20 extended Mexican American families collected as part of the San Antonio Family Studies. Imputation for missing SNP genotypes in pedigrees was conducted using a likelihood-based method implemented in MERLIN, based on the framework of available high-density genome-wide SNP data [22]. The GAW19 conveners simulated 200 replicates of phenotypic data based on the observed longitudinal data in the family-based sample. These data included 3 predicted deleterious coding variants in *CYP3A43* with simulated gene–medication interactions in the absence of genetic main effects. We were aware that carriers of these risk variants were assigned to be nonresponsive to the simulated BP treatment effect on SBP of −6.2 mm Hg (β_Int_ = 6.2 mm Hg). Additionally there were 984 SNPs with simulated genetic main effects for SBP explaining between less than 0.1 % and 2.78 % of the phenotypic variance.

### Accounting for family relatedness and population structure

We accounted for family relatedness using linear mixed models using a Comprehensive Mixed Model Program for Analysis of Pedigree and Population Data (MMAP) [23, 24]. To account for population structure, we applied principal component (PC) analysis to the observed genotypic data [21]. PCs were initially calculated in unrelated founders *(n* = 117) and a subset of 28,156 SNPs were selected for uniform coverage and low mutual linkage disequilibrium (r^2^ ≤ 0.2). PCs were assigned to all other individuals (*n* = 959) using estimated PCs from founders and the *predict* function in R to compute each individual’s PC scores based on the individual’s genotypes (www.R-project.org). We included the top 5 PCs in all association analyses [25].

### Gene–environment interaction analyses

Our analysis evaluated simulated SBP at the last time point (t = 3), when both the prevalence of hypertension and use of antihypertensive medications were the highest [1]. We assessed the appropriateness of model-based SEs by examining the heterogeneity of residuals by medication status using a likelihood ratio test to compare the homogeneity model with the heterogeneity model, and based on these results, our models allowed the residual error term to differ by medication status (*p* ≥0.05). All G × E analyses were adjusted for age, sex, population structure, and relatedness. We filtered out any SNP that exhibited a minor allele count of less than 2. The characteristics of true-positive findings were investigated at the 3 SNPs in *CYP3A43* with simulated gene–medication effects (1DF and 2DF tests), and the only SNP in *MAP4* with a simulated genetic main effect (2.87 % variation in SBP, chr3: 48040283, −9.91 mm Hg per minor allele) and greater than 80 % estimated power using a 2DF test. The FPP was calculated for the odd-numbered chromosomes using SNPs beyond 500 kb of the simulated effects (gene–medication effects for the 1DF tests, or simulated main and interaction genetic effects for the 2DF joint tests). Both true and false positives were considered statistically significant using a *p* value criterion of less than 0.05. Based on the simulated prevalence of medication status and SBP distribution, we estimated the expected TPP using an approximate effective sample size of 80 % of the total sample *(n* = 849), to account for the nonindependence of relative pairs. Power analyses were conducted using Quanto [26].

### “Interaction model”

The interaction between the simulated genotypes and BP medication status on SBP at t = 3 (equation 1) was modeled to calculate the estimated interaction effect, model-based SEs, and *p* values using MMAP [23]. The 1DF and 2DF joint tests (shown below) for this method have been described by Manning et al. [18].

#### Model 1

$$ {\mathrm{SBP}}_{\mathrm{t}=3} = \alpha + {\beta}_{{}_{{}_{SNP}}}{\mathrm{X}}_{\mathrm{SNP}} + {\beta}_{Med}{\mathrm{X}}_{\mathrm{Med}} + {\beta}_{Int}{\mathrm{X}}_{\mathrm{Int}} + {\beta}_C{\mathrm{X}}_C + g + e $$

Where X_Int_ = 1 when both X_SNP_ and X_Med_ are nonzero, C represents adjustments for covariates (age, sex, PCs), *g* ~ *N*(*σ*_*a*_^2^ *R*) is a polygenic random effect to account for familial correlation through a relationship matrix, and *e* ~ *N*(*σ*_*e*_^2^ *I*) is the error term. The null hypotheses for X_1DF_ and X_2DF_ are that *β*_*Int*_ = 0, and *β*_*Int*_, *β*_*SNP*_ = 0 jointly.

$$ {X}_{1df}=\frac{\beta_{Int}^2}{Var\left({\beta}_{Int}\right)}\sim {\chi}^2(1) $$

$$ {X}_{2df}={\left[\begin{array}{c}\hfill {\beta}_{SNP}\hfill \\ {}\hfill {\beta}_{Int}\hfill \end{array}\right]}^T{\left[\begin{array}{cc}\hfill Var\left({\beta}_{SNP}\right)\hfill & \hfill Cov\left({\beta}_{SNP},{\beta}_{Int}\right)\hfill \\ {}\hfill Cov\left({\beta}_{SNP},{\beta}_{Int}\right)\hfill & \hfill Var\left({\beta}_{SNP}\right)\hfill \end{array}\right]}^{-1}\left[\begin{array}{c}\hfill {\beta}_{SNP}\hfill \\ {}\hfill {\beta}_{Int}\hfill \end{array}\right]\sim {\chi}^2(2) $$

### Medication-stratified, “med-diff” approach

To apply the med-diff approach to BP medication–stratified results (Models 2a, b), we modeled the genetic main effect within strata of BP medication status using MMAP [23]. Then the Spearman rank correlation coefficient between strata for all SNPs (*r*, range across replicates and chromosomes: −0.13 to 0.16), magnitude, SE, and *p* value of the difference were estimated using EasyStrata [27]. The 1DF and the 2DF joint tests (shown below) have been described by Randall et al. [19] and Aschard et al. [28].

#### Model 2a

$$ {\mathrm{SBP}}_{\mathrm{t}=3} = \alpha + {\beta}_{SNP\Big|Med=1}{\mathrm{X}}_{\mathrm{SNP}\Big|\mathrm{M}\mathrm{e}\mathrm{d}=1} + {\beta}_C{\mathrm{X}}_C + g + e $$

#### Model 2b

$$ {\mathrm{SBP}}_{\mathrm{t}=3} = \alpha + {\beta}_{SNP\Big|Med=0}{\mathrm{X}}_{\mathrm{SNP}\Big|\mathrm{M}\mathrm{e}\mathrm{d}=0}+{\beta}_C{\mathrm{X}}_C + g + e $$

Where C represents adjustments for covariates (age, sex, PCs), and *g* and *e* are as in Model 1. The null hypotheses for Z_1DF_ and X_2DF_ are that *β*_*Diff*_ = 0, and *β*_*SNP|Med=1*_ *, β*_*SNP|Med=0*_ = 0 jointly.

$$ {Z}_{1df}=\frac{\beta_{\left(SNP\Big|Med=1\right)} - {\beta}_{\left(SNP\Big|Med=0\right)}}{\sqrt{S{E}_{\left(SNP\Big|Med=1\right)}^2 + S{E}_{\left(SNP\Big|Med=0\right)}^2-2rS{E}_{\left(SNP\Big|Med=1\right)}S{E}_{\left(SNP\Big|Med=0\right)}}}=\frac{\beta_{Diff}}{S{E}_{Diff}} \sim N\left(0,1\right) $$

$$ {X}_{2df} = \frac{\beta_{SNP\Big|Med=1}^2}{S{E}_{SNP\Big|Med=1}^2} + \frac{\beta_{SNP\Big|Med=0}^2}{S{E}_{SNP\Big|Med=0}^2}\sim {\chi}^2(2) $$

## Results

The average age was 48.1 years (58 % female) with the youngest and oldest participants across all replicates being 18 and 101 years old, respectively. The mean prevalence of BP medication use at the last simulated time point was 32.7 % (range across replicates: 30.2 to 36.4 %). The average change in SBP in individuals who initiated BP medication between the first and last time points was −6.9 mm Hg. The average odd-numbered chromosome-wide convergence was higher for the interaction model than for the med-diff approach (1DF: 8.0 × 10^6^ vs. 7.0 × 10^6’^, 2DF: 6.5 × 10^6^ vs. 5.7 × 10^6^).

### *CYP3A43* true-positive gene–medication effects (1DF)

**Fig. 1:**
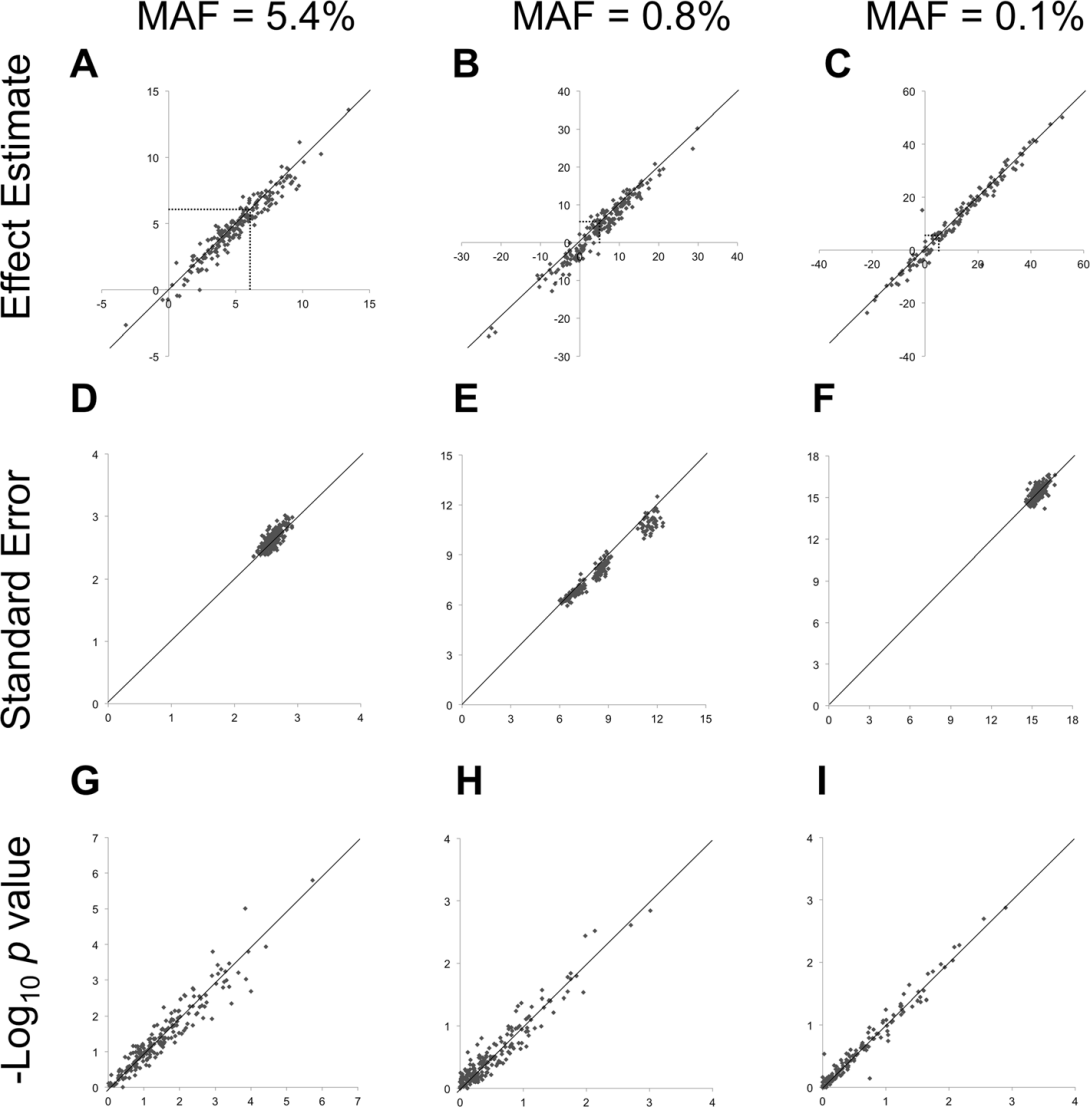
Comparison of the estimated effects (**a–c**), standard errors (**d–f**), and -log_10_ of the 1DF *p* values (**g–i**) on SBP from the interaction model (*x*-axis) and med-diff (*y*-axis) approaches in up to 200 replicates of simulated gene–medication interactions at 3 SNPs at *CYP3A43* (6.2 mm Hg, dashed line in (**a–c**) of varying minor allele frequencies (MAFs)

At the 3 SNPs at this locus the same replicates converged in both G × E approaches (Table 1)*.* Nonconvergence, caused by multicollinearity between the SNP and interaction terms or the small stratified-sample size, increased as MAF decreased for both approaches. The estimated interaction effects were slightly larger on average for the interaction model (difference 0.26–1.10 mm Hg) compared to the med-diff approach (Table 1). Both approaches produced estimated effects that varied across replicates, with the least-common SNP (Fig. 1a–c) showing the most variability in effect size. As compared to the simulated interaction effect of 6.2 mm Hg, the med-diff approach estimated this effect more conservatively on average than the interaction model, which resulted in less bias using the med-diff approach for the rarest of the 3 SNPs (MAF 0.1 %, Table 1) and more bias for the other 2 SNPs (MAF 0.8 % and 5.4 %). As would be expected, the estimated mean SE decreased as the SNP became more common (Fig. 1d–f). Yet at the least-common SNP with the poorest model convergence (MAF 0.1 %), the interaction and med-diff SE estimates were less variable than another more frequent SNP at the same locus (0.8 %, Table 1). Estimates of the 1DF and 2DF *p* values between the 2 approaches were comparable (Table 1, Fig. 1g–i). The TPPs identified using 1DF or 2DF tests were higher for the med-diff approach when the SNPs were less than 1 % MAF, but higher or equivalent for the interaction model when the SNP was common (MAF 5.4 %). The observed TPPs were not statistically significantly different from what we had estimated prior to the study (*p* ≥0.2) with the exception of the least-common SNP (*p* <0.02), which also did not converge for 30 % of the replicates (59 of 200 replicates).

**Table 1:** Power to detect true-positive gene–medication interactions at *CYP3A43* using 2 approaches. Gene–medication interactions (200 replicates) were simulated to be 6.2 mm Hg at 3 single nucleotide polymorphisms (SNPs) representing a range of minor allele frequencies (MAFs) (0.1 to 5.4 %)

	Interaction model				Med-diff approach			
	β_Int_	SE_Int_	1DF P_Int_	2DF P_SNP, Int_	β_Diff_	SE_Diff_	1DF P_Diff_	2DF P_Joint_
chr7:99457518-A (0.1 % MAF)								
Mean	12.35	15.42	4.3E-01	3.4E-01	11.82	15.29	4.2E-01	3.3E-01
Min	−21.90	14.55	1.3E-03	2.9E-04	−23.68	14.18	1.3E-03	6.1E-05
Max	51.85	16.69	1	9.9E-01	50.11	16.64	1	9.9E-01
Median	12.45	15.40	3.9E-01	2.4E-01	10.57	15.26	3.8E-01	2.3E-01
Replicates *P* <0.05 of 141 converged			12.1 %	17.0 %			13.5 %	18.4 %
chr7:99454482-G (0.8 % MAF)								
Mean	5.20	8.61	4.5E-01	5.0E-01	4.10	8.25	4.3E-01	4.9E-01
Min	−23.10	6.00	1.0E-03	2.3E-03	−24.83	5.96	1.4E-03	3.9E-03
Max	29.80	12.37	1	1	30.15	12.51	9.9E-01	9.9E-01
Median	5.45	8.37	4.5E-01	5.1E-01	4.76	7.88	4.2E-01	5.2E-01
Replicates *P* <0.05 of 190 converged			7.9 %	6.3 %			9.5 %	5.8 %
chr7:99457605-C (5.4 % MAF)								
Mean	5.11	2.60	1.3E-01	7.5E-02	4.85	2.63	1.5E-01	7.3E-02
Min	−3.20	2.30	1.9E-06	8.2E-06	−2.67	2.36	1.6E-06	2.8E-07
Max	13.44	2.92	9.8E-01	7.0E-01	13.59	3.02	9.9E-01	8.9E-01
Median	4.97	2.60	5.6E-02	2.2E-02	4.82	2.62	6.4E-02	2.0E-02
Replicates *P* <0.05 of 200 converged			47.5 %	66.0 %			43.5 %	66.5 %

*β* Effect estimate, *DF* degrees of freedom, *Diff* Difference, *Int* interaction, *Joint* Joint estimates of both interaction and main genetic effects, *MAF* minor allele frequency, *P p* value, *SE* standard error

### *MAP4* true-positive main genetic effects (2DF)

At chromosome (chr) 3:48040283, the main genetic effect was overestimated by both the interaction model (−13.1 mm Hg) and the med-diff stratified analysis of nonmedicated individuals (−13.3 mm Hg), whereas the med-diff analysis of medicated individuals underestimated the effect (−7.8 mm Hg). The 2DF joint *p* values were comparable between the 2 approaches with TPPs of 100 % *(n* = 156).

### False-positive proportions (1DF, 2DF)

**Fig. 2:**
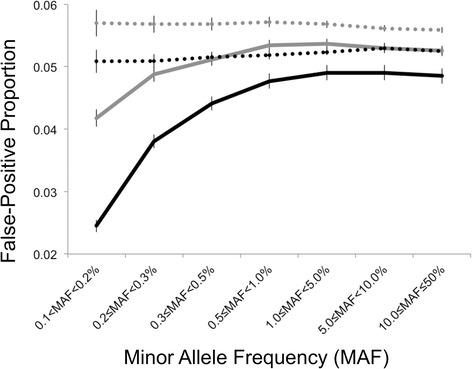
Comparison of the false-positive proportions (FPPs) and 95 % confidence intervals for the interaction model (solid line) and med-diff approaches (dashed line) and 200 replicates of true negative findings on the odd-numbered chromosomes using the 1DF (gray) and 2DF tests (black) across bins of minor allele frequency (>0.1 to 50 %). Note: False-positive proportions were calculated after excluding SNPs at the *CYP3A43* locus (chr7:98957518 to 99957518)

As shown in Fig. 2, the interaction model produced deflated FPPs in both the 1DF and 2DF tests, which approached 5 % across bins of increasing MAF. In contrast the med-diff approach produced stable FPPs across bins of MAF for both 1DF and 2DF tests. Both models produced higher FPPs for 2DF tests as compared to 1DF tests.

## Discussion

Randall et al. [19] have argued that 1DF tests, such as the interaction model and the med-diff approach, are particularly useful for informing public health interventions by highlighting the nature by which the environment may attenuate or exacerbate genetic predisposition to disease susceptibility. Our preliminary results thus far indicate that these 2 G × E approaches have notable differences with respect to the interaction effect simulated herein. The interaction model may be better at detecting true positive interactions than the med-diff approach for common SNPs, but the med-diff approach may estimate interaction effects more conservatively (i.e. closer to the null) and with less variability at rare SNPs (MAF <1 %). However, it is unclear how nonconvergence may be influencing these results.

The simulated GAW19 data set used in this analysis did not contain any simulated interaction effects at loci with simulated main genetic effects. Thus, using the simulated GAW19 data we were unable to validate a true positive with both main and interaction effects for either approach (2DF test), which may limit the generalizability of our findings. We were however able to compare our ability to detect a strong main effect at *MAP4* using a 2DF joint test of main and interaction effects. Even though we expect that a 1DF test of the main genetic effect would be a more powerful approach than a 2DF test when there is a true genetic main effect, this “true positive” assessment may represent a real world application of a 2DF test, wherein the investigator has no knowledge of the underlying true effects and may be interested in assessing the influence of potential interactions on established genetic loci. Furthermore, unlike the med-diff approach 1DF test implementation, the published 2DF test implemented in publicly available software does not account for the potential for correlation between strata due to relatedness. It is unclear how this may bias the observed 2DF results and methods comparisons made herein. Future work warrants a more thorough investigation of these approaches in family-based and unrelated data sets to detect associations at loci with both true main and interaction effects.

Based on our findings, the use of these G × E approaches on SNPs with less than 1 % MAF may lead to unstable TPPs and FPPs. First, nonconvergence may plague the most moderate samplings of the data, allowing the SEs to appear smaller than they really are and TPPs higher than expected, as we had observed for the least common *CYP3A43* SNP (MAF 0.1 %). At the SNPs examined at *CYP3A43* and *MAP4* we observed identical convergence between the 2 approaches. Yet we observed lower odd-numbered chromosome-wide convergence for the med-diff approach than the interaction model, because all medication-stratified models must have converged in order to apply the med-diff approach. Second, we observed FPPs for the interaction model less than expected (5 %) for both 1DF and 2DF tests, which was not the case for the med-diff approach. An analytic focus on low-frequency SNPs (MAF 1–5 %) or common SNPs (MAF >5 %) may minimize the observed difference in the FPPs between the 2 approaches.

## Conclusions

In this specific simulated example of gene-medication interactions in family-based data the med-diff approach exhibited greater power to detect interaction effects for low-frequency variants (MAF <1 %), whereas the interaction model exhibited greater power for common alleles (MAF >5 %). However, the med-diff method resulted in a stable but greater FPP for low-frequency variants as compared to the interaction method. In summary, both approaches are robust for common variants (MAF >5 %), but become less concordant as MAF decreases. One of the benefits of the stratified analysis of the med-diff approach is that it is less computationally intensive, but may not be appropriate for continuous environmental factors. This indicates that model selection may in part be context-specific. Furthermore if rare variation is of interest in future investigations, our findings here suggest that the med-diff approach may estimate true interaction effects more robustly, with less variability, and with stable FPPs around 5 % as expected; however, if common variants are the focus, the interaction model may be more robust.

Future investigations should consider different types of simulated interactions, for example, where genetic effects are in opposite directions in the 2 environmental strata or where genetic effects differ in magnitude between strata (ie, when both interaction and main effects are present). This current study used the GAW19 simulated family-based data to fill a gap in the G × E literature and make quantitative comparisons directly relevant to model choice in future effect estimation (TPP) and discovery (FPP) studies in the field of genetic epidemiology.
